# Decision-making strategies: ignored to the detriment of healthcare training and delivery?

**DOI:** 10.1080/21642850.2013.854706

**Published:** 2013-11-18

**Authors:** Chris Desmond, Kathryn A. Brubaker, Andrew L. Ellner

**Affiliations:** ^a^ Department of Global Health and Social Medicine, Harvard Medical School, Boston, MA, USA; ^b^ Human and Social Development Research Programme, Human Sciences Research Council, Pretoria, South Africa; ^c^ Division of Global Health Equity, Department of Medicine, Brigham and Women's Hospital, Boston, MA, USA

**Keywords:** decision-making, decision theory, health behavior, choice behavior

## Abstract

*Context*: People do not always make health-related decisions which reflect their best interest – best interest being defined as the decision they would make if they carefully considered the options and fully understood the information available. A substantial literature has developed in behavioral economics and social psychology that seeks to elucidate the patterns in individual decision-making. While this is particularly relevant to healthcare, the insights from these fields have only been applied in a limited way. To address the health challenges of the twenty-first century, healthcare providers and healthcare systems designers need to more fully understand how individuals are making decisions. *Methods*: We provide an overview of the theories of behavioral economics and social psychology that relate to how individuals make health-related decisions. The concentration on health-related decisions leads to a focus on three topics: (1) mental shortcuts and motivated reasoning; (2) implications of time; and (3) implications of affect. The first topic is relevant because health-related decisions are often made in a hurry without a full appreciation of the implications and the deliberation they warrant. The second topic is included because the link between a decision and its health-related outcomes can involve a significant time lag. The final topic is included because health and affect are so often linked. *Findings*: The literature reviewed has implications for healthcare training and delivery. Selection for medical training must consider the skills necessary to understand and adapt to how patients make decisions. Training on the insights garnered from behavioral economics and social psychology would better prepare healthcare providers to effectively support their clients to lead healthy lives. Healthcare delivery should be structured to respond to the way in which decisions are made. *Conclusions*: These patterns in decision-making call into question basic assumptions our healthcare system makes about the best way to treat patients and deliver care. This literature has implications for the way we train physicians and deliver care.

## 1. Introduction

Imagine that when making choices patients coolly and calmly evaluated every option available to them and, after considering all relevant information, selected the option that they determined to be best. Healthcare delivery would be straight forward. Providers would present relevant information and patients would choose the best course of action. Healthcare systems would need focus only on recommendations for healthy behaviors and screening tests and disease identification and treatment, with no concern for how patients may be making decisions that are not good for their health.

In actuality, decision-making is complicated, with different decision-making strategies being employed in different circumstances. People may be rushed and take mental shortcuts. Their emotions may cloud their judgment. They may be overly optimistic and fail to realistically evaluate risks. A failure to recognize this complexity can lead to healthcare which is inefficient, possibly ineffectual, and in some instances harmful. Social psychology and behavioral economics provide an increasingly clear picture of when people will use one or another strategy, but relevant insights from these subjects are largely ignored in the training of healthcare professionals, leaving them with only a lay understanding of what is the most important determinant of health – individual behavior (World Health Organization, 2011). Studies in social psychology and behavioral economics have highlighted numerous instances where an appreciation of how people are making decisions would benefit those who seek to influence such decisions, including healthcare providers. For example, a bias for the status quo and how if a healthcare provider makes a certain option the default, patients will more likely opt for that option, even if they are free to choose others (Halpern, Ubel, & Asch, 2007).

Individual behaviors are influenced by an array of social, political, cultural, and physical factors – many of which are largely out of individuals' control. There is extensive evidence in the epidemiological and biological literature that factors such as where someone lives, their place in a social hierarchy and whether they were abused in early childhood can put them at dramatically higher risk for all-cause morbidity and mortality, including chronic diseases heavily influenced by behaviors (Beaulac, Kristjansson, & Cummins, 2009; Diez Roux et al., 2001; Dube et al., 2003; Felitti et al., 1998; Malmstrom, Johansson, & Sundquist, 2001; Marmot, Shipley, & Rose, 1984; Marmot & Smither, 1991; Pickett & Pearl, 2001). However, within groups at high risk for behavior-related premature morbidity and mortality there are circles of people who nevertheless make healthy choices and overlapping circles of people who have unexpectedly good health outcomes. That some people make healthy choices within a high-risk context highlights the importance of understanding how such decisions are made and asking if it is possible to encourage and support more people to do the same.

Decision science should be thought of as a “basic science” of healthcare delivery. Healthcare providers should be trained in this basic science to improve efficiency and effectiveness of care. Just as microbiology and virology lead to biomedical advances, insights from the decision sciences lead to better approaches to providing care and counseling to individual patients.

In 1993, Redelmeier, Rozin, and Kahneman (1993), writing in *JAMA*, outlined ways in which intuitive decision-making may lead patients to take decisions which are bad for their health. They went on to argue “that an awareness of how people reason is an important clinical skill that can be promoted by knowledge of selected past studies in psychology” (p. 73). We argue the same, and ask why, 20 years later, decision-making does not receive the attention it deserves in medical training. Medical training is now lagging behind popular literature. Insights from social psychology, and increasingly from behavioral economics, are no longer confined to academic journals, but are the subject of popular books: for example, Kahneman's (2011) *Thinking, fast and slow* or *Nudge* by Thaler and Sunstein (2008).

Recent literature has reinforced the points made by Redelmeier et al. (1993). Swindell, McGuire, and Halpern (2010), for example, provide examples of the ways in which knowledge of decision strategies should change the ways physicians interact with and present information to patients. While we agree with the arguments made, we believe that the implications are not fully appreciated. We argue here that it is inappropriate to view a better understanding of decision-making as simply a potentially beneficial addition to healthcare; rather it should be seen as a central concern for healthcare training and delivery. Techniques such as motivational interviewing which help individuals choose healthier behaviors (Burke, Arkowitz, & Menchola, 2003) should not be seen as specialized methods, but rather as central approaches, and students should be provided with the theoretical underpinnings so they are able to appreciate the importance of such methods. Furthermore, we stress that a superficial understanding of decision-making will not be enough. As mentioned previously, individuals' behavior is heavily influenced by the context in which they live. To appreciate how context and decision-making strategies may be interacting requires a significant engagement with both sets of literature.

Our focus is on patients, but it is obvious that healthcare providers themselves may also be applying mental shortcuts or other decision-making strategies which are not in the best interest of the patient. The potential for physicians to be influenced by cognitive biases and heuristics has also received recent attention (Croskerry, 2013). System designers need to keep in mind that the patient examples we provide could equally apply to providers.

This paper is concerned with how healthcare providers and systems, and in particular primary care providers and systems, can influence general lifestyle decisions, such as those related to diet and exercise. In this paper we highlight a number of decision strategies which have been identified in the literature. This brief sampling of the literature provides a powerful support to the argument that ignoring these insights in healthcare training and delivery is ill-advised.

## 2. Insights into health-related decision-making

It is important to draw a distinction between when an individual makes a decision which may be bad for their health because they do not understand the available information and when they make such a decision because they are applying a particular decision-making strategy. Misunderstandings associated with low levels of literacy and numeracy are common and a source of concern (Kutner, Greenberg, Jin, Paulsen, & White, 2006; Lipkus, Samsa, & Rimer, 2001; Williams, Baker, Honig, Lee, & Nowlan, 1998; Woloshin, Schwartz, Moncur, Gabriel, & Tosteson, 2001). Misunderstandings have been linked to non-adherence to medical advice, and as a result of non-adherence, to poor health outcomes (Estrada, Martin-Hryniewicz, Peek, Collins, & Byrd., 2004; Gazmararian, Williams, Peel, & Baker, 2003; Kalichman, Ramachandra, & Catz, 1999; Schillinger et al., 2002). Numeracy skills in particular hinder individuals' ability to weigh health-related risks, at times leading to poor choices (Schwartz, Woloshin, Black, & Welch, 1997). Moreover, literacy and numeracy have been linked to difficulties with day-to-day health-related decision-makings, such as interpreting nutritional information (Peters, Hibbard, Slovic, & Dickmann, 2007; Rothman et al., 2006). Misunderstandings are important and training for healthcare providers on when to expect them and how to address them is potentially very useful. However, even without training, healthcare providers are likely to be aware that misunderstandings are a problem; whereas they may not be aware of the implications of different decision-making strategies.

The decision-making strategy employed by an individual depends on, among other things, how much time they have, their circumstances and characteristics, the information that is available to them, their emotional state and the type of decision being made (Kahneman, 2011). To highlight the importance of training healthcare providers in decision science we examine three (overlapping) topics, namely: (1) mental shortcuts and motivated reasoning; (2) implications of time; and (3) implications of affect. The topics were selected as they provide powerful examples of decision-making strategies which are relevant to healthcare providers, particularly those providers wishing to encourage behavior change or promote informed decision-making. Mental shortcuts are particularly relevant in the healthcare setting. Brief episodic encounters with healthcare providers may generate a pressure for a quick decision. Moreover, healthcare providers' actions may unintentionally influence the outcome of that decision. Motivated reasoning is surely a frustration for many providers, when despite the provision of information to the contrary their patients continue to base their actions on a previously formed conclusion. The consequences of health-related decisions are rarely instantaneous and often occur long after an interaction with a healthcare provider seeking to support that individual. How individuals are considering the passage of time in their decision-making is, therefore, critical. Affect, the final topic, can be heavily influenced by healthcare providers, particularly through their framing of decisions. Given the implications of affect on decision-making, an awareness of this mechanism is arguably critically important for healthcare providers. These topics are intended as examples and do not provide a definitive summary of the relevant aspects of the social psychology and behavioral economics literatures. They are intended to highlight why these literatures are relevant to healthcare training and delivery.

### 2.1 Mental shortcuts and motivated reasoning

People do not always (or even often) carefully consider the costs and benefits of every option available to them; time constraints or the perception that the decision is not a critical one can lead individuals to apply mental shortcuts (heuristics) or stick with the status quo (Kahneman, Knetsch, & Thaler, 1991). As a result, even when they understand the information provided to them, people make decisions (including health-related decisions) that may be contrary to their considered desires. The benefit gained from spending additional time on a decision may or may not be worth the effort. The key is to identify when heuristics or other shortcuts are being applied to decisions which warrant more careful consideration. Training healthcare providers on the common mental shortcuts will help them identify when their patients may be applying them to health-related decisions which warrant more consideration. Examples of common heuristics and shortcuts include the representativeness heuristic, the anchoring heuristic and the status quo bias (Kahneman, 2011).

Predicting when the representativeness heuristic may be being applied, for example, is potentially critical. This heuristic is applied when individuals are considering the likelihood that an uncertain event will occur. Rather than focus on available statistical information such as the estimated probability of the event or the sample size on which that estimate is based, individuals consider more easily understood information, namely how similar to or representative of other known situations the uncertain event is (Tversky & Kahneman, 1974). The representativeness heuristic is often applied when individuals are estimating their risk of contracting an illness. Gerend, Aiken, West, and Erchull (2004) investigated women's perception of their risk of breast cancer, congestive heart failure, and osteoporosis. They found that the extent to which a woman perceived herself to be similar to the average woman who contracted one of those diseases accounted for a large amount of the variance in perceived susceptibility. If healthcare providers assume that a patient is basing their decisions on the statistical information they have provided them to assess their risk, they may miss that the patient is under- or overestimating risk because they consider themselves either dissimilar or similar, respectively, to the stereotype they have of a person who suffers for the condition being discussed.

Healthcare providers similarly need to be aware of the anchoring heuristic which occurs when people make estimates about uncertain events by assimilating information they already have. Anchoring is problematic when they assimilate irrelevant information (Epley & Gilovich, 2006). For example, a random number mentioned at the outset of an interview has been seen to influence people's answers to a range of questions from the length of the Mississippi to the weight of Caesar (Furnham & Boo, 2011). Klein and Stefanek (2007) note that in the conversations physicians have with patients about personal risk, anchors could come from a variety of statements not related to the topic at hand. Moreover, patients use their subjective risk as an anchor and will not adjust their perceived risk sufficiently even after being provided with information concerning their objective risk. Senay and Kaphingst (2009), for example, report that counseling for women who overestimated their risk for breast cancer failed to lower risk estimates to realistic levels. Again, a failure to recognize that this is occurring could result in a healthcare provider incorrectly assuming that a patient is making a decision based on a realistic assessment of risk.

Perhaps the most straight forward shortcut that healthcare providers should be aware of is the status quo bias – the tendency to choose what is seen as the default, even if it is not the best option (Samuelson & Zeckhauser, 1988). Halpern et al. (2007) discuss several ways that the status quo bias could be used to influence individual decisions relating to health. For example, to increase rates of participation, organ and cadaver donation, pneumococcal vaccination for hospitalized patients, and HIV screening during regular check-ups could all be opt-out rather than opt-in.

Lessons from decision science not only help identify when shortcuts are being applied, but also suggest approaches to minimize their application. It has been shown that individuals will spend more time and energy reasoning if they are motivated to be accurate (Kunda, 1990). Similarly when they believe their judgments will be made public or that they will have to justify them, they are less likely to use anchoring or stereotypes (Freund, Kruglanski, & Shpitzajzen, 1985).

An awareness of mental shortcuts can aid in the identification of opportunities within the healthcare setting to assist patients in making the decision that best represents that patient's desires and values. Moreover, if healthcare providers understand their shortcuts they can try and identify when in day-to-day life individuals are applying them to decisions that have important negative implications for their health and work to find ways to support them to do otherwise.

### 2.2 Implications of time

Understanding decisions which require considering costs and benefits at different points in time is important in the context of healthcare, and in particular primary care, as outcomes of decisions relating to health typically play out over time. Exercise, healthy eating, smoking, utilizing healthcare services, adhering to drug regimens, and many other behaviors all require the person deciding to undertake (or not) such a behavior to consider the consequences for their future. A failure to understand these processes will often lead to missed opportunities to promote healthy lifestyles, and as a result, to inefficient care.

Numerous studies have shown that the majority of individuals do not have time consistent preferences, i.e. they discount highly in the short term, but soon start to be relatively indifferent between longer and longer waits. Behavioral economists refer to this as hyperbolic discounting. Evidence of hyperbolic discounting has been observed in a variety of contexts (Cairns & van der Pol, 2000; Simpson & Vuchinich, 2000), including various models of animal preferences (Freeman, Green, Myerson, & Woolverton, 2009; Richards, Mitchell, Wit, & Seiden, 1997). Hyperbolic discounting is a concern for healthcare providers as it may affect the reliability of patients' stated intentions. A patient may state that next week they will start exercising after work before going home to watch television. However, when next week comes, the cost of delaying television watching is seen as too high and they go straight home. The patient is not lying, their preferences are simply time inconsistent. This dynamic is often apparent with problem drinking (Vuchinich & Simpson, 1998).

Time inconsistent preferences also arise when individuals have difficulty predicting how a visceral state will affect their decision-making. Such state dependent preference switching is referred to as the “hot–cold empathy gap” and has been documented in numerous contexts. Nordgren, van der Pligt, and van Harreveld (2008) found that dieters and smokers in satiated states were more likely to set ambitious quitting or weight loss goals for themselves, while those who were currently hungry or craving set more modest goals. Similarly it has been found that those in a sexually unaroused state underestimate what they would do to receive sexual gratification, which satiated opioid addicts underestimate the strength of their cravings, and that prior to labor, women underestimate their desire for an epidural (Ariely & Loewenstein, 2006; Badger et al., 2007; Chistensen-Szalanski, 1984).

Healthcare providers who are not aware of the tendency for individuals to have time inconsistent preferences may adopt different strategies to those who are aware. When a patient does not follow through on a stated intention the unaware provider may assume that the patients are simply lying or not committed enough to the behavior. Aware providers could explain time inconsistent preferences to their patients and together work on strategies to address the problem; for example, working through “what if?” scenarios with the patient.

Similarly, aware healthcare providers could work with patients to help them identify other predictive shortcomings such as the optimism bias. This bias refers to the well-documented phenomenon that individuals frequently underestimate their risk of experiencing negative life and health events. Clarke, Lovegrove, Williams, and Machperson (2000), for example, surveyed women and men about their estimates of breast and prostate cancer risk; both groups demonstrated an optimism bias relating to their perceived risk, benefits of screening, and years surviving after diagnosis. Providers must, however, be cautious not to over correct for optimism, a pessimistic outlook leading to depression is hardly an appropriate goal.

To identify when the optimism bias may be in play, it is useful to understand where it stems from. A variety of sources have been identified. Klein and Helweg-Larsen (2002), for example, suggest that the greater amount of control an individual believes they have over an event or situation, the less they estimate their personal risk to be. Weinstein (1987) emphasizes that individuals tend to extrapolate from past experience to estimate future risk, concluding that if the problem has not yet appeared, one is exempt from future risks. Perloff and Fetzer (1986) found that when asked to make comparisons between themselves and others, and the target was left sufficiently vague, individuals tended to construct downward comparisons so that they could perceive themselves as less vulnerable. This motivational aspect of the optimism bias, of wanting (even if only at a subconscious level) to perceive oneself as less vulnerable, is especially apparent in studies carried out with smokers. A survey of community college students, for example, found that 42% of smokers believed that continuing to smoke would hurt their health only a little, if at all, and 45% believed that quitting would provide little if any health benefit (Prokhorov et al., 2003).

Although people tend to be too optimistic about the likelihood that negative events will not occur, they tend to be pessimistic about how well they would cope with them should they occur. When asked to predict what their quality of life would be while suffering a specific illness, healthy individuals often estimate a lower quality of life than that which is reported by actual sufferers. Sackett and Torrance (1978) found that on the health-related quality of life scale (0 being death and 1.0 being perfect health), dialysis patients reported a .56 while healthy patients estimated a .39. Using the same scale, patients without colostomies estimated a .8 while patients with colostomies reported a .92 (Boyd, Sutherland, Heasman, Tritchler, & Cummings, 1990). These discrepancies have been attributed to healthy individuals not anticipating the ways in which they would successfully adapt to poorer health. Indeed, by focusing study participants' attention on the ways in which they could adapt to a chronic illness or disability, the size of the discrepancy between quality of life estimates of healthy and sick individuals is seen to decrease (Ubel, Jepson, & Loewenstein, 2005).

In the health domain, decisions and outcomes are often separated by a substantial amount of time. The way in which patients consider time in their decision-making is therefore central to health-related decision-making. Understanding how people incorporate time, and in particular how this can lead to apparently inconsistent preferences over time, is critical to supporting patients to make, and follow through with, healthy decisions.

### 2.3 Implications of affect

People's judgments can be influenced by the emotional state they are in at the time they make a decision. The implications of this range from switching preferences based merely on how a problem is framed to how people understand and respond to risk. Being aware of the ways in which these factors can influence decision-making can help healthcare providers think through how and when options are presented. If providers are unaware, they may be inadvertently promoting a particular choice, which may or may not be to the benefit of the patient.

Loss aversion is a powerful emotional response which can affect decision-making – people are more sensitive to losses than they are to equivalent gains (Tversky & Kahneman, 1981). Loss aversion helps explain why negative or positive framing of potential health outcomes can influence the treatment options that individuals select. For example, when participants in a study were provided with the prognostic outcome information for an infant born at 23 weeks framed as either survival with lack of disability (positive) or chance of dying and likelihood of disability (negative), those participants given the gain-framed messages were more likely to elect resuscitation over comfort care (Haward, Murphy, & Lorenz, 2008). Rothman and Salovey (1997) found that people were more likely to carry out illness detection behaviors when the messaging they received was loss framed. It has been suggested that loss aversion occurs because individuals underestimate their ability to rationalize loss and overestimate their tendency to dwell on it and so are willing to engage in riskier behavior to avoid it (Kermer, Driver-Linn, Wilson, & Gilbert, 2006).

In addition to being aware of loss aversion, it is important to consider the implications of a decision-maker's affect on their perception of risk. Johnson and Tversky (1983) induced feelings of negative or positive affect in study participants and then asked them to estimate the frequency of various fatalities. They found that participants who had induced negative affect were more likely to perceive the various causes of death as more likely than those who had positive affect induced. A study carried out after the 11 September terrorist attacks induced either fear or anger in participants regarding those events and then asked them to rate the likelihood of events related to the attacks such as whether the USA would capture Osama bin Laden. Those participants in the anger condition provided more optimistic responses than those in the fear condition (Lerner, Gonzalez, Small, & Fischhoff, 2003).

Healthcare providers are often faced with having to deal with emotional topics and with patients in emotional states. Moreover, providers often have control over how options are framed. Anticipating the importance of framing and affect can help providers think through the best ways to support patients.

The cursory review of the above three topics suggests that contrary to being perfectly rational, individuals may be misinformed, biased, and/or inconsistent when making decisions related to their health. The notion that people coolly and rationally weigh the known, quantified risks and benefits of different courses of action, as argued in the economic theory of rational choice, does not hold empirically (Kahneman, 2011). When viewed in light of the central role that individual health-related decisions play in determining health outcomes, these findings challenge some of the most basic tenets of how health providers interact with patients and by implication, of how future providers are prepared for the practice of medicine.

## 3. Implications for healthcare training

As a result of many factors, but, in particular, the highly influential Flexner Report on medical education, US medical training in the twentieth century came to focus almost exclusively on biomedical science, with consideration of human behaviors, psychology, and interactions included as, at best, an afterthought. Until very recently, premedical requirements and testing were exclusively focused on the “hard” or “basic” sciences of chemistry, physics, and organic chemistry. Likewise, the primary curriculum in the pre-clinical years of medical training came to be built around anatomy, biochemistry, physiology, and pathophysiology, with courses on patient interviewing and social sciences fitted in around the margins. In clinical training, with the notable exception of Family Medicine programs, hospital-based medicine predominates, with much briefer exposure to ambulatory and community-based healthcare.

A health workforce with such a narrow focus on biomedical science in its selection and training will be ill-prepared to meet the health needs of its future patients and of society. This is not to suggest that healthcare should be grounded in anything but the most rigorous possible empirical, scientific basis; but rather that a health workforce well-prepared to address the key drivers of premature morbidity and mortality would have training that balances, with equal sophistication and emphasis, social with biomedical science. This would suggest fairly sweeping changes which we will only briefly touch on here, including the need to select candidates for healthcare training with substantive preparation and demonstrated competence in the social sciences and emotional as well as intellectual intelligence; to re-structure pre-clinical and clinical training to include robust and rigorous training in human systems and social sciences, particularly in fields such as social psychology and behavioral economics, but also in organizational behavior and systems engineering; and to reform graduate medical training so that it is focused on preparing a workforce that delivers a high-quality, high-value service to patients and society – likely one less focused on hospital medicine and rescue care and more focused on prevention and behavior change. Such changes in training may not be needed across the broad range of healthcare providers. Those involved in purely technical aspects of healthcare, such as laboratory technicians, are not in as great a need of understanding decision-making as those who engage directly with patients or design programs and systems which shape patients' environments. Doctors, nurses, counselors, and system designers are the primary targets for revised training.

The good news is that these types of changes have already begun. The American Association of Medical Colleges recently announced that the Medical College Admission Test will be restructured so that nearly half its emphasis is on social and behavioral sciences and critical readings that emphasize questions of ethics and cross-cultural studies. Innovative medical schools and programs, with approaches that integrate biomedical and social science and community-based, multidisciplinary approaches into their core curriculum, have sprung up around the country. This transformation could be hastened by changes in the design of graduate medical education funding to enable and incentivize further innovations in medical training and to produce trainees capable of integrating biomedical and social science and of leading the high-value healthcare delivery systems of the future.

## 4. Implications for healthcare delivery

Insights from decision science should influence the re-design of healthcare delivery and the practice of medicine as we move toward systems that achieve better health outcomes at lower cost. The current paradigm, which relies largely on brief, episodic interactions between providers and patients and the straightforward, static presentation of probabilistic information about the risks and benefits of different courses of action understandably and manifestly fails to support patients' healthy choices.

A person's health is determined through a complex, adaptive process. While traditional approaches to healthcare have predominantly focused on the safety and efficacy of individual, often short-term interventions (diets, medications, surgical procedures, and other behavioral interventions), decision science suggests that individuals do not think and act in a rational, linear way – and, even if they did, they would be subject to large-scale changes beyond their immediate control in their sociopolitical and physical, external environment, which profoundly impact their health. Real-world effectiveness in healthcare delivery will be greatly enhanced by a systems approach that focuses on achieving outcomes through dynamic and diverse processes. Providers and healthcare delivery systems should work with patients to identify measurable, personally meaningful goals to work toward together, rather than focusing only on agendas of specific interventions for specific disease processes, prioritized largely by providers and the structures and incentives built into the healthcare system. Some examples of how to make this shift are given below.

### 4.1 Improve longitudinal relationships and communication between patients and providers

The current paradigm of predominantly visit-based interactions between patients and providers is not well-suited to how people make decisions about their health. Earlier a number of causes of time inconsistent preferences and inability to predict outcomes were discussed. Healthcare delivery systems must capitalize on the explosion of innovation in communication technologies to fundamentally change how providers and patients interact. Shorter, more frequent, asynchronous interactions (e.g. over email or even text message or Twitter) may more effectively support patients in their decision-making process and make more efficient use of providers' time. Models for healthcare organization and, particularly, reimbursement will need to change to accommodate this new communication paradigm. Asynchronous interactions should not replace all visit-based interactions as these are essential to relationship building. They do, however, provide options to strengthen support and increase the chance of behavior change, in a way that a visit only system does not.

### 4.2 Understand the emotional side of health decision-making

The evidence overwhelmingly suggests that people are influenced at least as much by emotions and affect as by intellectual reasoning when making decisions about their health. Being careful to never manipulate this reality, providers should balance their presentation of risks to patients with substantive appeals to emotions and not just to intellect. The power of different framings is substantial.

### 4.3 Focus on mental health

Given the effect of affect on decision-making and the high prevalence of mental illness, particularly among the chronically ill, we can no longer afford for sophisticated approaches to mental health to be the purview of a relatively small group of highly trained specialist providers. Mental healthcare must be integrated into all aspects of healthcare delivery and providers must be much more knowledgeable about mental illness and cognizant of the impact that an altered affect can have on all other aspects of patient care.

## 5. Conclusion

People are complex and this complexity is reflected in the decisions they make and the behaviors that result. If rational choice theory was correct and individuals carefully evaluated the costs, benefits, and risks associated with every option available to them, the work of healthcare providers would be simple. Healthcare providers would only need to focus on diagnosis and providing information on alternative responses. They would not need to be concerned that patients might be applying mental shortcuts and not giving the various options due consideration or selecting the default option because they think it is being recommended. They would not need to worry about how they frame options, as framing would make no difference. They would not be concerned that what the patient commits to do and what they will actually do will differ because of the hot–cold empathy gap.

Given the importance of behavior in determining health outcomes and the complexity of decision-making, it is critically important for healthcare providers to make use of the insights into this complexity which are available in the socio-psychology and behavioral economics literatures. The brief review of these literatures summarized here provides an indication of the types of insights on which a foundation of a fuller understanding of health-related decision-making can be built. This fuller understanding can, in turn, assist in the training of healthcare providers – better preparing graduates to support patients to make and maintain healthy decisions. Moreover, these insights can inform the re-design of healthcare systems to take greater advantage of interactions with patients.

The insights provided by these literatures are informative, but they are also limited. The literature points to when a certain decision-making strategy is more likely to be employed. This works well when explaining the distribution of decision outcomes across a population. The healthcare provider is often faced with an individual, not a population. Further work to identify predictors of decision strategies could make efforts to limit the use of inappropriate or harmful strategies more efficient.

What would also support the adaptation of these insights to the healthcare setting is to conduct research specifically focused on health-related decision-making. The interaction between approaches to healthcare provisions and application of heuristics and motivated reasoning in relation to health-related decisions would be particularly informative. The results of such research would create opportunities for further adjustments to provide training and healthcare delivery and hopefully for more effective and efficient systems of care. The potential use of technology to support the maintenance of healthy behaviors is already an area of research and should be supported. Finally, a critical area of research would be to test if the adaptations to training suggested in this paper will have the expected impact on the quality of healthcare provision.
